# Case of Gunshot Wound, and Subsequent Extraction of a Bullet from the Bladder

**Published:** 1850-08

**Authors:** E. M. Macpherson

**Affiliations:** Assistant-Surgeon 9th Lancers


*Case of Gunshot Wound, and Subsequent Extraction of a Bullet from the Bladder.* By E. M. Macpherson, Assistant-Surgeon 9th Lancers. (Proceedings of Royal Medical and Chirurgical Society, March 26, 1850.)—A private in H. M. 24th regiment was wounded (at the battle of Chillianwallah, on the 13th of January, 1849,) in the left buttock: severe pain was immediately felt in the testicle on the same side: the ball could not be found, but the wound healed without difficulty: no blood was ever noticed in the urine. Symptoms of disturbance of the bladder shortly afterwards set in, which not yielding to remedies the bladder was examined, and a foreign body detected; and on the 30th of August the lateral operation, as if for the removal of a calculous, was performed: an iron ball was extracted, which had become encrusted with a thin layer of sandy deposit. To the above case Mr. Dixon added notices from various writers of fifteen operations for the extraction of balls, which had either primarily entered the bladder, or having lodged in the immediate neighborhood, had made their way into its cavity. Mr. Dixon had been favored by Mr. Cusack, of Dublin, with a notice of a similar operation performed by him, and another by the late Mr. Colles, neither of which has been published: in three cases extraction was not attempted, or was unsuccessfully tried, the bullets, forming nuclei of stones, having been found in the bladder after death; in one case the bullet was small enough to be voided by the urethra. The situation of the external wound in the cases cited was very various. The time that elapsed between the infliction of the wound and the removal of the ball varied from a day or two to ten years. The lateral operation was performed in the majority of cases, but the high operation had been employed by Baudens on account of the ball having entered at the bottom of the linea alba, so that by enlarging the recent wound he could reach the cavity of the bladder.—*London Med. Gaz.* April 5, 1850.

